# Supplementary material for: Low-Field Benchtop NMR to Discover Early-Onset Sepsis: A Proof of Concept
Source: Metabolites. 2023 Sep 21;13(9):1029. doi: 10.3390/metabo13091029 (PMC10535760; doi:10.3390/metabo13091029)

Supplementary Materials

Figure S1. Relevant ROIs discovered by stability selection: the signals within the relevant ROIs are reported in red. Aligned LF NMR spectra are reported in the plot; the region of the water suppression between 4.50 ppm and 5.25 ppm was removed.

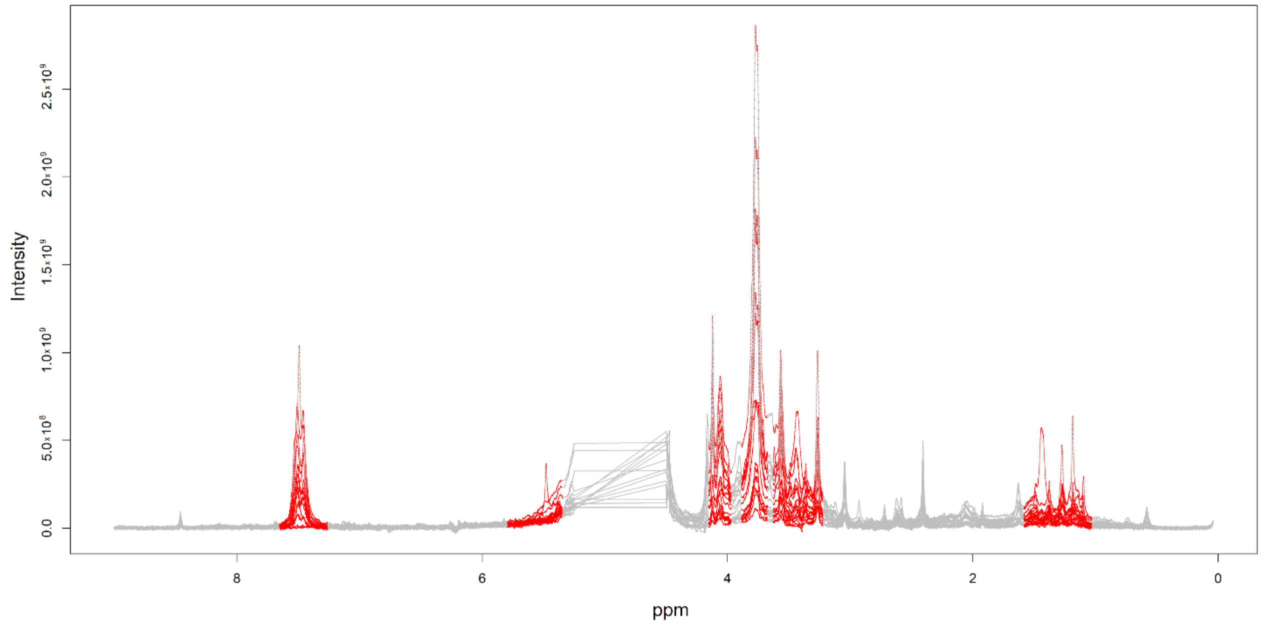

Supplement: Supplementary file 1 [file metabolites-13-01029-s001.zip › metabolites-2622560-supplementary.pdf]
